# The Relationship between Nocturnal Hypoxemia and Left Ventricular Ejection Fraction in Congestive Heart Failure Patients

**DOI:** 10.1155/2014/978358

**Published:** 2014-02-13

**Authors:** Mohammad Mirzaaghazadeh, Mehrzad Bahtouee, Fariba Mehdiniya, Nasrollah Maleki, Zahra Tavosi

**Affiliations:** ^1^Department of Internal Medicine, Imam Khomeini Hospital, Ardabil University of Medical Sciences, Ardabil 6368134497, Iran; ^2^Department of Internal Medicine, Shohadaye Khalije Fars Hospital, Bushehr University of Medical Sciences, Bushehr, Iran

## Abstract

Congestive heart failure (CHF) is a major cause of mortality and morbidity. Among patients with heart failure, sleep disordered breathing (SDB) is a common problem. Current evidence suggests that SDB, particularly central SDB, is more prevalent in patients with CHF than in the general population, but it is underdiagnosed as SDB symptoms that are less prevalent in CHF. The main aims of this study were to determine the relationship between nocturnal hypoxemia and left ventricular ejection fraction in patients with chronic heart failure. By means of echocardiography, 108 patients with left ventricular ejection fraction ≤45% were divided into mild, moderate, and severe CHF. Hypoxemia was recorded overnight in the hospital and was measured by portable pulse oximetry. In the 108 patients with CHF, 44 (40.7%) were severe, 17 (15.7%) moderate, and 47 (43.6%) mild CHF. 95 (88%) of patients with CHF had abnormal patterns of nocturnal hypoxemia suggestive of Cheyne-Stokes respiration. Ejection fraction correlated negatively with dip frequency. There was no correlation between nocturnal hypoxemia with BMI and snoring. This study confirms strong associations between sleep apnea and heart disease in patients with CHF. Overnight oximetry is a useful screening test for Cheyne-Stokes respiration in patients with known heart failure.

## 1. Introduction

Sleep related breathing disorders (SRBD) refer to an abnormal respiratory pattern (e.g., apneas, hypopneas, or respiratory effort related arousals) or an abnormal reduction in gas exchange (e.g., hypoventilation) during sleep. They tend to repetitively alter sleep duration and architecture, resulting in daytime symptoms, signs, or organ system dysfunction. Sleep related breathing disorders are best characterized by polysomnography that has captured one or more periods of rapid eye movement (REM) sleep, as severe perturbations can be common during REM sleep [1, 2]. Sleep apnea is hypothesized to increase the risk of developing cardiovascular disease (CVD) and hypertension. Initial support for this hypothesis came from several population studies of snoring and CVD outcomes, suggesting that those who snore are more likely to develop hypertension, myocardial infarction, and stroke [3–5]. Two types of sleep disordered breathing are common among patients with heart failure: obstructive sleep apnea (OSA) and Cheyne-Stokes breathing (CSB).

### 1.1. Prevalence

While OSA is more common than CSB in the general population, CSB may be more common than OSA in patients with heart failure [6, 7]. Single-center observational studies estimate that the prevalence of SRBD may be as high as 50 percent among all patients with heart failure and as high as 70 percent among patients with heart failure who are referred to a sleep laboratory [6–9]. The prevalence may be even higher among patients with acute decompensated heart failure, as suggested by a study that detected an apnea hypopnea index ≥10 events per hour of sleep in 22 out of 29 such patients (76 percent) [10].

### 1.2. Risk Factors

Risk factors for SRBD in patients with heart failure vary according to the type of SRBD. With respect to CSB, risk factors include male gender, advanced age, atrial fibrillation, and hypocapnia (i.e., transcutaneous carbon dioxide ≤38 mmHg) [9]. With respect to OSA, risk factors include advanced age and an increasing body mass index (BMI).

### 1.3. Pathogenesis

The pathogenesis of OSA involves abnormalities in pharyngeal anatomy, pharyngeal function, and ventilatory control. In patients with heart failure, edema of the upper airway is an additional factor that may contribute to pharyngeal airway narrowing [11]. The pathogenesis of CSB is uncertain, but the favored hypothesis is based on the observation that patients who have heart failure and CSB tend to have lower arterial carbon dioxide tensions (PaCO_2_) than patients who have heart failure without CSB [12, 13]. The net effect is oscillation of ventilation between apnea and hyperpnea. Elimination of the hypocapnia with inhaled CO_2_, continuous positive airway pressure (CPAP), or oxygen can markedly attenuate CSB [14–17]. Both OSA and CSB can impair systolic and diastolic cardiac function by a variety of mechanisms. First, intermittent hypoxemia and arousals induce adrenergic surges that may lead to heart disease progression. Second, the extremely negative intrapleural pressures increase ventricular transmural wall stress and afterload [18].

### 1.4. Clinical Manifestations

A sleep history should be sought from both the patient and the spouse because, in many cases, it is only the spouse who is aware of the abnormal ventilatory pattern. SRBD can be asymptomatic or symptomatic in patients who have heart failure [19]. When OSA is the predominant type of SRBD, poor sleep quality and snoring are common. As a result, sleep disruption and easy fatigability often exist and may be out of proportion to the severity of the heart failure. However, sleepiness is relatively uncommon in patients with heart failure for reasons that remain unclear [20]. When CSB is the predominant type of SRBD, symptoms due to CSB may be indistinguishable from those due to the heart failure [6]. Symptoms of poor sleep quality (e.g., excessive daytime sleepiness) are subtle and generally unreliable. Occasionally, patients with CSB report paroxysmal nocturnal dyspnea (due to the hyperpnea that follows an apnea) [21]. SRBD may contribute to nocturnal angina in patients with heart failure, presumably due to hypoxemia and catecholamine surges [21]. In addition, recurrent arrhythmias may occur, such as atrial fibrillation or ventricular tachycardia [9, 22]. These arrhythmias often occur in the absence of any symptoms or signs of SRBD. Thus, a high index of suspicion should be maintained and evaluation for SRBD should be considered in heart failure patients with recurrent arrhythmias.

### 1.5. Diagnosis

The diagnostic evaluation of suspected SRBD is the same for patients with or without heart failure. An in-laboratory overnight polysomnogram is the gold standard diagnostic test. In-home portable monitoring is also available. The 2005 American College of Cardiology/American Heart Association (ACC/AHA) guidelines on the diagnosis and treatment of chronic heart failure indicate that screening for SRBD is reasonable in selected patients (e.g., those with risk factors) [23].

### 1.6. Prognosis

Heart failure accompanied by SRBD is associated with a worse prognosis than heart failure in the absence of SRBD [24]. With respect to OSA, a prospective cohort study followed up 164 patients who had heart failure and a left ventricular ejection fraction of 45 percent or less [25]. At a mean of three years, patients who had OSA (defined as an AHI of at least 15 events per hour) had a higher cardiac mortality than patients who did not have OSA (8.7 versus 4.2 deaths per 100 patient-years). With respect to CSB, a prospective cohort study followed up 62 patients with NYHA class II to III heart failure [26]. At a mean of 28 months, cardiac mortality was associated with an AHI greater than 30 events per hour. The AHI was a better predictor of cardiac mortality than demographic variables, Holter monitoring, exercise studies, echocardiography, or autonomic testing. CSB was found to predict mortality in numerous other studies of patients with heart failure [7, 27–30].

### 1.7. Treatment

With respect to the impact of heart failure therapy on SRBD, case series and observational studies suggest that the following interventions are associated with improved SRBD: medical management (e.g., ACE inhibitors, beta blockers, and diuretics) [11, 25, 31, 32], cardiac transplantation [33–35], cardiac resynchronization (i.e., biventricular pacing) [36–38], and left ventricular assist device (LVAD) implantation [39]. For patients who have heart failure complicated by OSA or CSB, positive airway pressure may improve cardiac function, blood pressure, exercise capacity, and quality of life [15, 28, 40–46]. The possible role of theophylline in patients with heart failure complicated by SRBD was evaluated in a doubleblind crossover trial of 15 such patients who received either theophylline or placebo twice daily for five days [47].

## 2. Materials and Methods

The current cross-sectional study is a descriptive, analytical one that was conducted on 108 patients referred to the Imam Hospital from November 2010 to March 2011, who had been hospitalized due to CHF. CHF diagnosis was performed based on history, clinical examination, and echocardiography. Given the prevalence of heart failure as 14% in Iran and considering that, in accordance with previous studies, approximately 51% of these patients suffer from sleep disorders resulting from changes in arterial oxygen pressure, the sample size for this study was estimated as 108 patients. Inclusion criteria for this study included patients with systolic CHF (congestive heart failure) (EF less than or equal to 45%) and people with chronic obstructive pulmonary diseases (COPD) and patients with unstable CHF were excluded.

Information on age, sex, BMI, and sleep patterns of patients was obtained. Spirometric examinations were performed on patients with CHF, and patients with COPD diagnosis based on medical history, physical examination, and spirometry were excluded. Then, eligible patients underwent pulse oximetry from the night until the next morning using DC-68B (Shenzhen Creative Industry wrist oximeter).

In this study, the statistical software SPSS V16 was used for data analysis. The tests including chi-square, *t*-test, Pearson correlation coefficient, and ANOVA were used for obtained data analysis. The significance level was determined as less than or equal to 0.05.

## 3. Results

In this study, 108 patients with stable chronic heart failure were studied; 52 patients (48.1%) were males and 56 patients (51.9%) were females. The patients' age range was 35–86 years with a mean of 65.42 ± 11 years, and the patients, BMI was between 20 and 38 with a mean of 26.93 ± 3.74. 73 patients (67.6%) had a BMI greater than or equal to 25 and 35 patients (32.4%) had a BMI less than 25 (Table 1). Patients were studied regarding sleep disorders. 62 patients (57.4%) were snoring at night, and only 18 patients (16.7%) complained of daytime sleepiness. The patients were classified regarding ejection fraction into three groups, including mild failure (ejection fraction between 40 and 45 percent), moderate failure (ejection fraction between 35 and 40 percent), and severe failure (less than or equal to 34 percent of ejection fraction). The results showed that 47 patients (43.6%) had mild heart failure; 17 patients (15.7%) had moderate heart failure; and 44 patients (40.7%) were with severe heart failure.

The patients were also examined regarding the percentage of their nocturnal sleep duration suffered from hypoxia. The results showed that 42.6% of patients experienced hypoxia in 10% of their total nocturnal recording time; 9.1% of patients had suffered from hypoxia in 40–70% of their total nocturnal recording time; 5.4% of patients had hypoxia in more than 75% of their total nocturnal recording time; and in 12% of patients, hypoxia was not observed in total nocturnal recording time. During evaluating the patients about daily hypoxia (during waking hours), the results showed that only 6.5% of the patients experience arterial oxygen desaturation at the time of awakening.

Patients with different levels of ejection fraction and with different levels of arterial oxygen desaturation were compared (Table 2). The results showed that patients with mild heart failure in the majority of arterial blood oxygen saturation levels had the least hypoxia time; however, the analysis of variance showed no significant difference between the three groups of patients. Thus, Pearson correlation test was also performed to investigate the relationship between levels of arterial oxygen desaturation and ejection fraction. The results showed that, at levels of arterial oxygen desaturation between 80–84%, 75–79%, and 65–69%, there is a significant correlation between the two variables of arterial oxygen saturation percentage and ejection fraction, so that with decrease in ejection fraction, the arterial oxygen desaturation in patients increases.

The arterial oxygen desaturation was also calculated according to patients' gender. The results showed that arterial oxygen desaturation rate in women is higher than in men; however, performing *t*-test showed no statistically significant differences between the two groups (*P* = 0.43). The correlation test was also performed between arterial oxygen desaturation and the age of patients; however, no significant correlation was found between these two variables (*P* = 0.07).

Arterial oxygen desaturation in patients with complaints of sleep disorders and without sleep disorders was examined. The results showed that the decline in arterial oxygen saturation is higher in patients with nocturnal snoring than patients without snoring at night; however, performing independent *t*-test showed no significant differences between the two groups (*P* = 0.90). The mean arterial oxygen desaturation rate was also compared in two groups of patients with BMI greater than or equal to 25 and less than 25. The results showed that patients with a BMI greater than or equal to 25 have a greater mean arterial oxygen desaturation rate than patients with lower BMI; however, the independent *t*-test showed no significant differences between the two groups (*P* = 0.72).

## 4. Discussion

Heart failure (HF) is a major cause of mortality and morbidity [48] and is associated with progressively severe symptoms, chronic disability, and impaired quality of life [6]. Sleep-disordered breathing (SDB) is known to occur frequently in patients with stable but severe HF [6, 19, 48] and may be a predictor of poor prognosis [29]. Sleep related breathing disorders (SRBD) appear to be common even among patients whose heart failure is optimally managed. SDB is present in approximately three-fourths of patients with symptomatic or decompensated systolic heart failure [45, 46]. The prevalence is very high even in those with stable chronic heart failure [6, 47, 48]. Cross-sectional analyses from Sleep Heart Health Study data revealed an adjusted odds ratio of 2.2 for self-reported heart failure amongst subjects with OSA.

In this study, 88% of patients with heart failure experienced SRBD during their total nocturnal recording time. In a study by Javaheri et al. in 1998, 51% of male patients with stable heart failure had SRBD, 40% of which was central sleep apnea type (CSA) and 11% was obstructive sleep apnea type (OSA) [6]. In another study in China by Wang et al. conducted on 195 patients with heart failure, the SRBD was seen in 80% of patients, 53% of OSA type and 27% of CSA type [49]. In a study by Rao et al. performed in 2006 in the United Kingdom, the SRBD prevalence was reported on average as 24% [50]. In a study by Lanfranchi et al., the SRBD was found in 55% of patients with asymptomatic heart failure with LVEF less than 40% [19, 26]; but in a study by Tremel et al., 82% of patients with heart failure had SRBD [51]. A prospective cohort study followed up 108 patients who visited a heart failure clinic with stable heart failure, which was defined as clinical stability without hospitalizations or medication changes within the past 30 days [52]. SRBD was detected in 61 percent of patients and was independently associated with the presence of atrial fibrillation and a worse New York Heart Association (NYHA) functional class.

In the present study, no significant relationship was found between age and arterial oxygen desaturation. In a study by Staniforth et al. in 1998 performed on 104 patients, no significant relationship was found between age and arterial oxygen saturation. In this study, the nocturnal pulse oximetry was identified as a useful screening test for Cheyne-Stokes respiration in patients with heart failure [53]. However, in a study by Sin et al. to evaluate risk factors of CSA and OSA performed on 450 patients with heart failure, the patients with CSA were older than patients with OSA [9]. In a study by Liu et al. in 2006 on 56 elderly patients with CHF, 67.9% of patients had SRBD and the OSA prevalence was higher in older patients [54]. Male gender may be a risk factor for CSA because, in general, men have a less stable sleep architecture than women, with a greater number of sleep-wake transitions and shorter slow-wave sleep, which may predispose to respiratory control system instability and central apneas [55].

In our study, patients with a BMI greater than 25 had a mean arterial oxygen desaturation higher than patients with a lower BMI; however, no statistically significant difference was found between the two groups. Considering that polysomnography was not available in our study and evaluation of arterial oxygen desaturation was performed by using a portable pulse oximetry, there was no possibility to differentiate patients with OSA from CSA. However, since the prevalence of CSA in patients with cardiac failure is more than OSA, we expect that most of our patients have CSA, and the lack of significant correlation between BMI and arterial oxygen desaturation can be explained in this regard. In Javaheri et al.'s study, high BMI was more common only in patients with OSA [6]. In Sin et al.'s study, high BMI was significantly correlated with OSA. In this study, high BMI association with OSA, which was more in men, was due to android pattern of obesity in them [9].

In the present study, snoring at night (nocturnal snoring) was seen in 57.4% of patients. In heart failure patients who had nocturnal snoring, the mean arterial oxygen desaturation was more than in patients without this clinical symptom; but there was no statistically significant difference between the two groups. In Javaheri et al.'s study, 78% of patients with OSA had nocturnal snoring, and only 28% of patients with CSA had nightly snoring [6].

In our study, daytime sleepiness was seen in only 16.7% of the patients. In Wang et al.'s study, patients with heart failure who had SRBD were not mentioning daytime sleepiness [49]. The first goal in the treatment of SDB in CHF is to optimize CHF treatment. Conservative measures for OSA such as weight reduction, avoidance of supine position during sleep, and avoiding alcohol and sedative medications before sleep are also useful. Nocturnal CPAP therapy may be useful in treating SDB in CHF [56].

In the present study, there was a significant correlation between the severity of arterial oxygen desaturation rate and ejection fraction. In similar studies by Staniforth et al. [53], Javaheri et al. [6], and Shahar et al. [57], a significant correlation between decreased ejection fraction and arterial oxygen desaturation was seen.

## 5. Conclusion

Several studies in recent years have demonstrated the close relationship between sleep related breathing disorders (SRBD) and systolic heart failure. In the present study, 88% of patients with heart failure experienced SRBD during their total nocturnal recording time. Unfortunately, in clinical treatment of systolic heart failure, SRBD is hardly considered.

## Figures and Tables

**Table 1:** Distribution of patients according to age and body mass index.

Variable	Number	Minimum	Maximum	Mean	SD
Age	108	35	86	65.42	11.40
BMI (Kg/m^2^)	108	20	38	26.93	3.74

**Table 2:** Correlation between the two variables of arterial oxygen saturation percentage and ejection fraction in study patients.

Arterial oxygen saturation percentage	Mild CHF (mean ± SD)	Moderate CHF (mean ± SD)	Severe CHF (mean ± SD)	*P* value
60–65%	0.17 ± 0.41	0.11 ± 0.25	0.34 ± 0.65	0.16
65–70%	0.22 ± 0.58	0.11 ± 0.20	0.32 ± 0.47	0.31
70–75%	0.68 ± 1.75	1.73 ± 6.51	0.81 ± 1.13	0.42
75–80%	1.84 ± 6.09	3.85 ± 11.26	2.57 ± 3.69	0.57
80–85%	3.90 ± 8.11	7.23 ± 12.17	5.84 ± 8.75	0.36
85–89%	12.77 ± 15.52	14.02 ± 16.67	16.04 ± 18.40	0.65
